# Perturbations can distinguish underlying dynamics in phase-locked two-neuron networks

**DOI:** 10.1186/1471-2202-14-S1-P50

**Published:** 2013-07-08

**Authors:** Sharon E Norman, Carmen C Canavier, Robert J Butera

**Affiliations:** 1 School of Electrical and Computer Engineering, Georgia Institute of Technology, Atlanta, GA 30332, USA; 2 Department of Cell Biology and Anatomy, Louisiana State University Health Sciences Center, New Orleans, LA 70112, USA; 3 Neuroscience Center of Excellence, Louisiana State University Health Sciences Center, New Orleans, LA 70112, USA; 4 Department of Biomedical Engineering, Georgia Institute of Technology, Atlanta, GA 30332, USA

## 

Synchronization of neuronal activity is observed in high- and low-level functions of the nervous system. For example, during memory tasks, neural activity in different brain regions phase-locks [1,2], while synchronized cells in the brainstem contribute to respiratory function [3]. In a two-cell system, network phase is a measure of when one neuron spikes with respect to the other neuron, normalized by the network period; phase-locked systems have a constant network phase, and here we define synchronized systems as those with a network phase close to zero. Understanding how and under what conditions neurons synchronize and phase-lock is important for understanding how neuronal populations function.

Phase resetting curves (PRCs) describe how a neuron's period changes in response to inputs applied at various times during the interspike interval [4,5]. We use a PRC-based map of stimulus times vs response times [6] to predict if two coupled neurons will phase-lock, as well as how robust this phase-locking is against perturbations; two curves, one per neuron, are plotted against each other on this map, and intersections of the curves correspond to fixed points of the coupled system. Stable fixed points indicate stable phase-locking, while unstable points predict movement around the map. Close, but non-intersecting, curves result in networks that show a preferred phase with some phase slips. The fixed points (or lack thereof) on this map determine the dynamics of the coupled system, but similar statistics of network phases can be obtained from different underlying dynamics. Our goal is to discern underlying dynamic properties of the coupled system when a PRC cannot be measured.

Here we explore perturbation-based methods to distinguish between different fixed point cases that result in similar network phase histograms. Because fixed points determine the network's response to perturbations, we use perturbations to uncover if, and where, fixed points exist without actually creating the PRC-based map. Parameters for coupled, conductance-based neuron models are chosen such that different numbers of fixed points produce phase histograms with similar network phases. When a synaptic perturbation or random noise is applied to one simulated neuron, the resulting trajectory of subsequent cycles around the map plane gives clues to the location and presence of underlying fixed points; different trajectories that point to the same location indicate a stable fixed point, while trajectories that point away from a region indicate an unstable point. Trajectories that only traverse the map plane in one direction are indicative of zero fixed points but close curves. Because biological neurons are not perfectly regular oscillators, we also investigate the effects of noise on the network phase histogram and how it affects our ability to resolve fixed point cases. The simulation results presented here will be validated in experimental hybrid circuits of one *Aplysia californica *neuron and one computational neuron coupled using the dynamic clamp.
